# Oropharyngeal Tularemia: A Case From Rural Armenia Highlighting Diagnostic Challenges and Treatment Outcomes

**DOI:** 10.7759/cureus.84439

**Published:** 2025-05-19

**Authors:** Fiza Khan, Md Foorquan Hashmi, Goulia Ohan, Vigen Asoyan, Alvard Hovhannisyan

**Affiliations:** 1 Department of General Medicine, Yerevan State Medical University after Mkhitar Heratsi, Yerevan, ARM; 2 Department of Epidemiology, Yerevan State Medical University after Mkhitar Heratsi, Yerevan, ARM; 3 Department of Infectious Diseases, Yerevan State Medical University after Mkhitar Heratsi, Yerevan, ARM

**Keywords:** armenia, case report, elisa, francisella tularensis, oropharyngeal tularemia, tularemia

## Abstract

Tularemia, a life-threatening zoonotic infection caused by *Francisella tularensis*, is a significant public health concern. Diagnosis can be challenging due to nonspecific symptoms, including fever, tonsillitis, and lymphadenopathy. We present the case of a 48-year-old male from a rural area of the Tavush region admitted to our hospital, initially presenting with left-sided tonsillitis, followed by the development of enlarged lymph nodes in the neck and a mild fever. Despite initial treatment with amoxicillin, tularemia was confirmed through positive serological tests, including antibody titers of 1:400 by the volumetric agglutination method and elevated IgM (0.74) and IgG (0.51) levels by enzyme-linked immunosorbent assay (ELISA). Treatment with ciprofloxacin was initiated, leading to the resolution of fever but subsequent lymph node suppuration. Prompt drainage and a course of doxycycline resulted in patient healing and discharge. This case underscores the importance of considering tularemia in patients presenting with tonsillitis, fever, and lymphadenitis, particularly in endemic and rural areas, and emphasizes the necessity of timely diagnosis and early treatment to prevent complications and economic burdens.

## Introduction

*Francisella tularensis* is a facultative intracellular, Gram-negative bacterium responsible for tularemia, also known as "rabbit fever," a zoonotic disease. The infection is primarily contracted through contact with infected wildlife, particularly hares, or via arthropod vectors such as ticks and deer flies. Additional modes of transmission include inhalation of contaminated aerosolized particles and ingestion of contaminated food or water. The bacterium typically enters the human body through direct contact with infected animal tissue or exposure of mucous membranes [1].

*Francisella tularensis*, the etiologic agent of tularemia, exhibits high virulence in both human and animal hosts, capable of effective transmission at low infectious doses due to its aerosolized form. Notably, there is no documented evidence of person-to-person transmission. Due to its high infectivity and stability in aerosol form, *F. tularensis *is classified as a select agent. The clinical presentation of tularemia varies depending on the route of infection [2]. Following exposure, the incubation period ranges from three to six days, with acute illness potentially persisting for several weeks. The most commonly reported symptoms include fever, headache, arthralgia, myalgia, ulcers, dyspnea, sweating, weight loss, and ocular irritation, with conjunctivitis occurring if the infection originates in the eye [1].

The clinical manifestations of tularemia are influenced by factors such as the infectious dose, inoculation site, and strain virulence. The most prevalent form of the disease, ulceroglandular tularemia, is characterized by an ulcer and regional lymphadenopathy at the inoculation site and can persist for several months. Consumption of contaminated food or water can result in oropharyngeal or gastrointestinal tularemia. The most severe form, pneumonic tularemia, arises either from inhalation of aerosolized bacteria or as a complication of other disease forms. The preferred pharmacological treatments for tularemia are streptomycin and gentamicin [3]. Although tetracycline and chloramphenicol are frequently used, they have been associated with treatment failure. While tularemia is fatal in approximately 5% of untreated cases, the mortality rate is reduced to less than 1% with appropriate antibiotic therapy. The disease is geographically widespread across the Northern Hemisphere, with endemic foci identified in Eastern Europe, Canada, the United States, Japan, and certain regions of China [4]. The distribution of tularemia is non-homogeneous, influenced by various environmental factors such as climate, host and vector population density, and the susceptibility of different host species to infection [5].

In the Republic of Armenia, where tularemia is endemic in approximately 95% of the territory, the disease poses a significant public health concern. Given that *F. tularensis* is a select agent, administrative regions (marzes) are required to submit monthly reports on human cases to the Centers for Disease Control and Prevention (CDCP) in Yerevan [3]. Serological testing remains the primary diagnostic method. During the Soviet era, mass vaccination campaigns targeted residents in areas with natural foci of the disease. Although a live vaccine strain (LVS) for tularemia exists, its use remains limited [6].

This report presents the case of a 48-year-old male presenting with generalized weakness and cervical lymphadenopathy. He was admitted to the Infectious Disease Department of the Mikaelyan Surgical Institute, where serological analysis confirmed the presence of tularemia-specific antibodies.

## Case presentation

**Figure 1:**
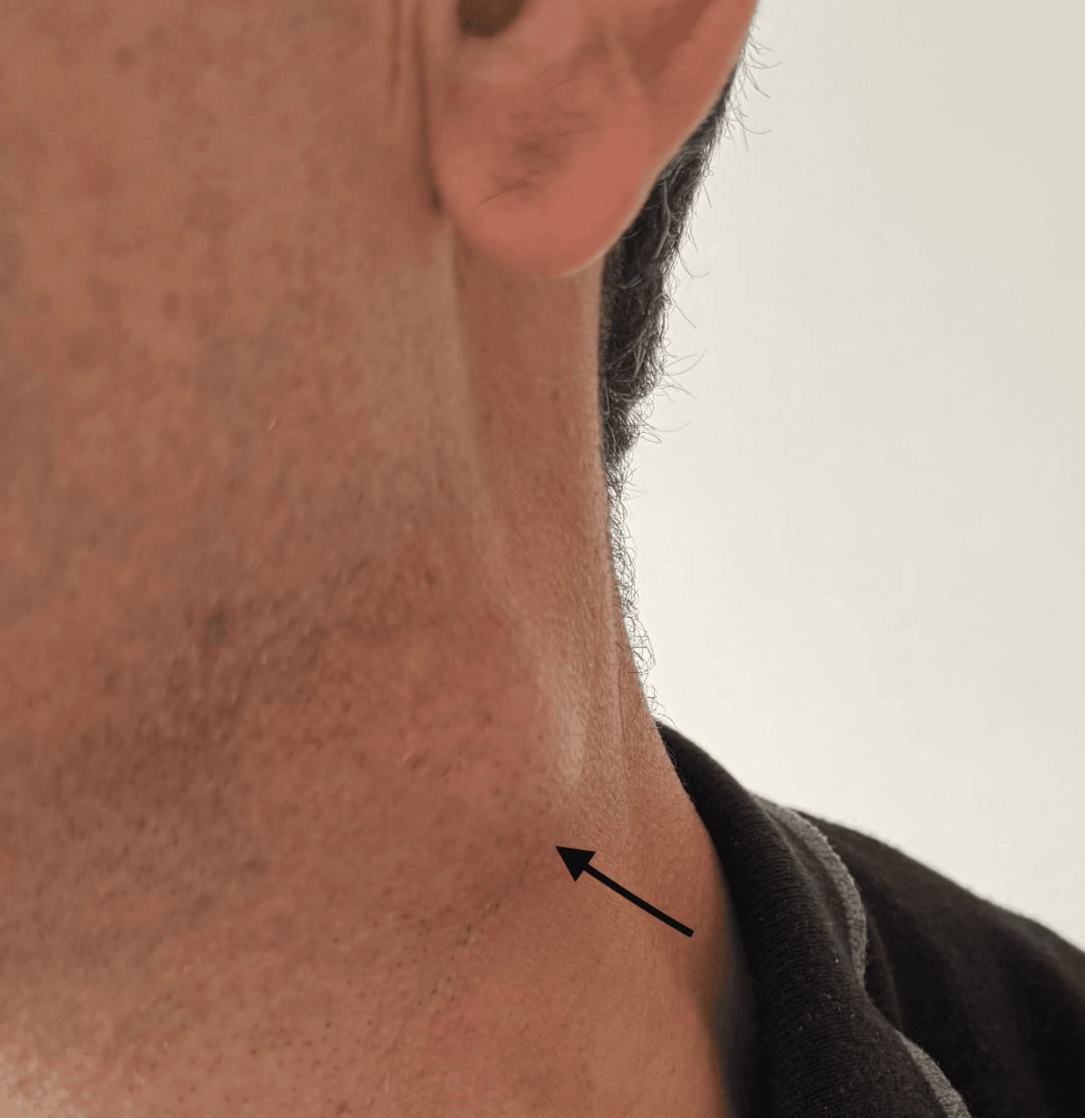
Enlarged cervical lymph node (arrow).

A 48-year-old male from the rural Tavush Region in Armenia presented with a two-month history of feeling unwell. He initially experienced left-sided tonsillitis, for which he received antibiotic therapy (amoxicillin) with partial improvement. Approximately 10-12 days later, he noticed an enlarging lymph node in the left neck area (Figure 1), prompting medical attention.

On examination, tenderness was noted over a movable lymph node, approximately the size of a chicken egg (diameter ≈ 3cm), in the left submandibular area. Additionally, a ≈2 cm diameter, movable, mildly tender lymph node was palpated in the lower anterior cervical region, with no visible skin changes. The oropharyngeal mucosa was hyperemic.

Tests for hepatitis B surface antigen (HBsAg), human immunodeficiency virus antigen/antibody (HIV Ag/Ab), hepatitis C virus antibody (HCV Ab), and Syphilis Ab were negative. Laboratory investigations revealed elevated levels of immunoglobulin M (IgM) (0.74) and immunoglobulin G (IgG) (0.51). Serologic testing for *Francisella tularensis* revealed elevated antibody levels, with a titer of 1:400 detected through the volumetric agglutination reaction (VAR) method (Table 1). Imaging studies, including an abdominal CT scan, demonstrated inhomogeneous fluid formations in the left neck region, with subsequent scans showing an inhomogeneous fluid mass.

**Table 1:** Laboratory investigations of the patient. IgM: immunoglobulin M; IgG: immunoglobulin G; VAR: volumetric agglutination reaction; HBsAg: hepatitis B surface antigen; HIV Ag/Ab: human immunodeficiency virus antigen/antibody; HCV Ab: hepatitis C virus antibody

Test	Result	Reference Range
HBsAg	Negative	Negative
HIV Ag/Ab	Negative	Negative
HCV Ab	Negative	Negative
Syphilis Ab	Negative	Negative
IgM against* F. tularensis*	0.74	<0.40
IgG against *F. tularensis*	0.51	<0.30
Antibody titer (VAR)	1:400	<1:100

Diagnosis of tularemia with cervical lymphadenopathy was made based on the clinical presentation, serological findings, and imaging results. Upon further inquiry, it was revealed that the patient had noted the presence of rats in his basement, where food is stored.

Treatment was initiated with ciprofloxacin 400 mg twice daily for 14 days, supplemented with anti-inflammatory therapy. The patient's condition significantly improved during hospitalization, and he was discharged with instructions for completing the full course of antibiotics.

**Figure 2:**
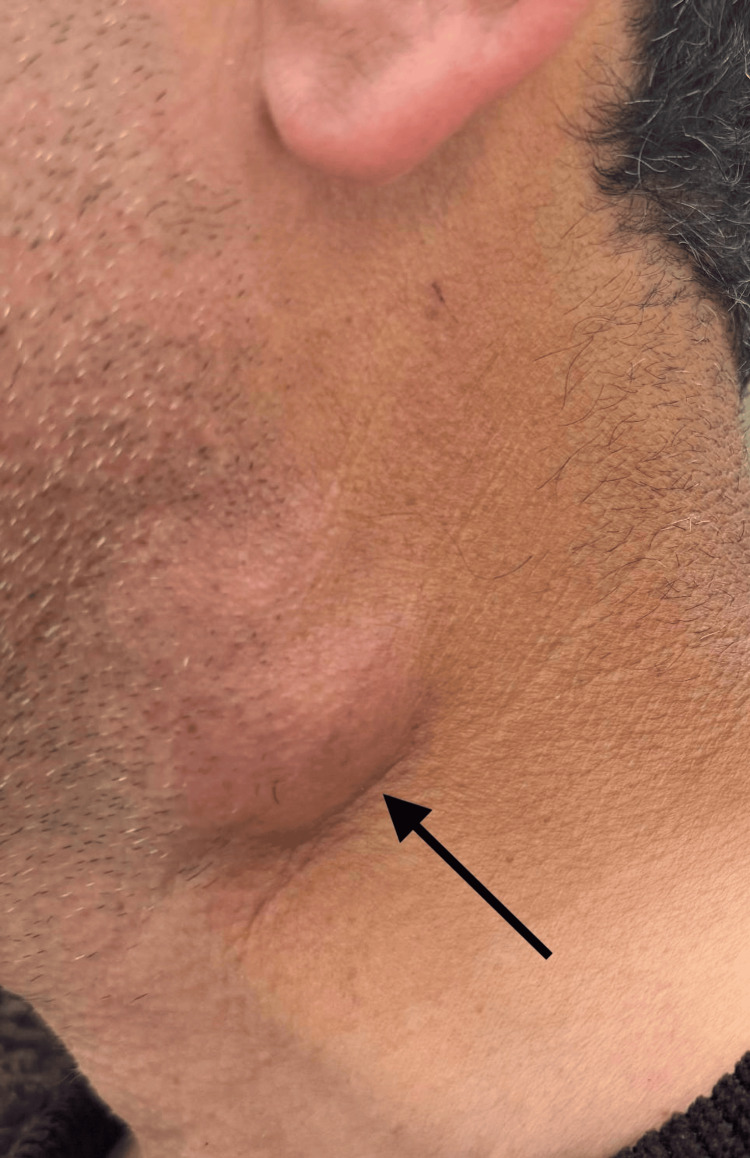
Painful, suppurated cervical lymph node (arrow).

**Figure 3:**
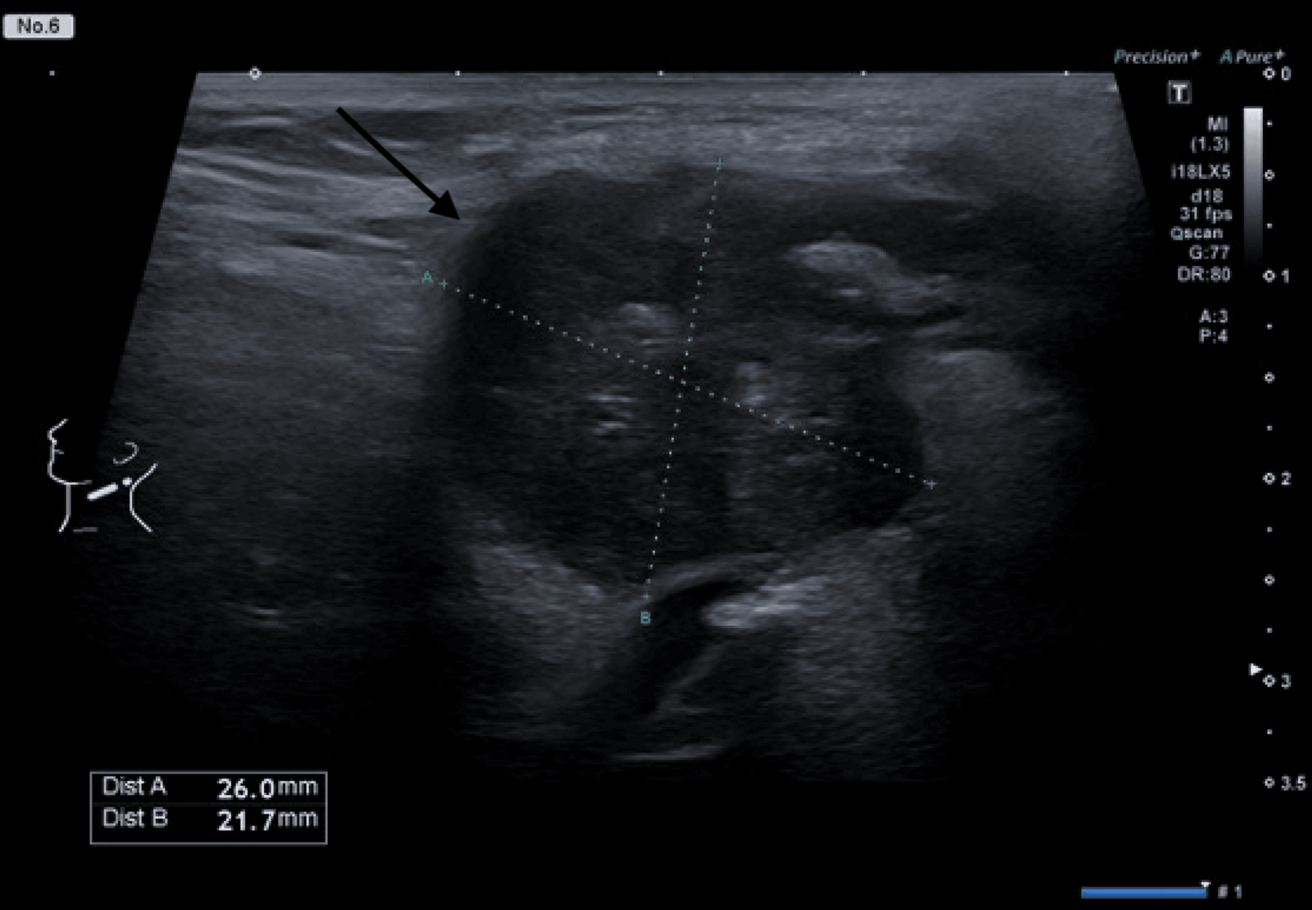
Ultrasound showing 2.6 x 2.2 x 2.0 cm (arrow) affected lymph node.

After 21 days of treatment, the patient had no fever, but suppuration of lymph nodes was observed (Figure 2). An ultrasound was performed on the lymph node, which revealed a liquid consistency (Figure 3). The decision was made to drain the suppurative lymph node and prescribe doxycycline for seven days, after which the patient healed and was discharged from the hospital.

## Discussion

Tularemia, caused by the bacterium *Francisella tularensis*, is a zoonotic disease with various clinical manifestations depending on the route of transmission and host factors [7,8]. The most common form of tularemia is ulceroglandular, marked by an ulcer at the site of infection and swelling of nearby lymph nodes [8]. However, the alimentary route of infection, as seen in this case, is one of the most common in Armenia [9] and can lead to the oropharyngeal form of tularemia, which presents with tonsillitis, fever, and cervical lymphadenitis [10]. 

Between 1996 and 2012, Armenia documented 266 instances of tularemia in humans. When categorized by clinical presentation, the cases were divided as follows: glandular form accounted for 69% (182 cases), angino-bubonic form for 3% (eight cases), and oculoglandular form for 28% (75 cases) [11]. Subsequent research by M. Davidyants et al., spanning from 2000 to 2015, revealed a shift in distribution, with bubonic cases being the most common at 49.1% (n=28), followed by oropharyngeal cases at 36.9% (n=21), and oculoglandular cases at 14% (n=8) [9]. In our case, the patient was diagnosed with tularemia with anginal bubonic form. 

*F. tularensis* is categorized as a Class A biowarfare agent due to its high infectivity rate, stability in liquid environments, ease of growth, rapid spread, and capacity to cause severe illness and morbidity. Symptoms usually appear within two to nine days after exposure. The oropharyngeal form typically manifests with symptoms such as pharyngitis, stomatitis, and cervical lymphadenopathy [8].

Oropharyngeal tularemia should only be diagnosed after excluding ulceroglandular tularemia. This distinction can be difficult to make, especially when the infection is acquired through mosquito or tick bites to the head or neck. In such cases, patients may present with enlarged cervical lymph nodes but no visible skin ulcer. An epidemiological investigation into the patient's food and water sources can provide clues to oropharyngeal tularemia [12]. In our case, the patient did not recall any history of a skin ulcer but mentioned the presence of rats in his basement, which raised suspicion for the oropharyngeal form.

In cases of nonspecific lymphadenopathy, various potential differential diagnoses should be considered, including streptococcal or staphylococcal infections, brucellosis, bartonellosis, sporotrichosis, infections caused by *Pasteurella multocida*, and parotitis associated with mumps [13,14]. Moreover, the presence of symptoms such as fever and overall malaise, along with localized swelling of lymph nodes, might suggest the possibility of lymphoma [14,15].

Laboratory testing played a crucial role in confirming the diagnosis. Serologic assays, such as the volumetric agglutination test and enzyme-linked immunosorbent assays (ELISAs) for IgM and IgG antibodies against F*. tularensis*, are commonly used for diagnosis [8,16]. In this case, positive serology supported the clinical suspicion of tularemia, prompting appropriate antibiotic therapy with ciprofloxacin.

The diagnosis of tularemia is often delayed unless it is specifically considered, which can result in the unnecessary use of multiple antibiotics and an increased risk of complications, such as lymph node suppuration. In some cases, surgical intervention may be required [17]. In our case, although the fever resolved with ciprofloxacin therapy, the patient developed lymph node suppuration that required additional intervention. Ultrasound imaging confirmed the presence of purulent material within the lymph node, prompting drainage and a switch to doxycycline therapy. The timely drainage of the abscess decreases the necessity for intravenous antibiotic administration and surgical removal of the lymph node [14].

This case underscores the significance of public health awareness and surveillance in endemic regions. Early recognition of tularemia cases can facilitate prompt treatment, reducing the risk of complications and transmission within communities. Clinicians practicing in endemic areas should educate patients about preventive measures, such as avoiding contact with sick or dead animals, wearing protective clothing during outdoor activities, and promptly seeking medical attention for febrile illnesses.

## Conclusions

Tularemia is endemic in Armenia, presenting a significant public health concern. Our case illustrates the varied clinical presentations of tularemia and the importance of considering this diagnosis in patients with febrile illnesses and lymphadenopathy, particularly in endemic regions. Prompt recognition and treatment are crucial to prevent complications and transmission within communities. This case also emphasizes the need for public health awareness and surveillance measures to mitigate the impact of tularemia outbreaks. 
